# Mitophagy, Mitochondrial Dynamics, and Homeostasis in Cardiovascular Aging

**DOI:** 10.1155/2019/9825061

**Published:** 2019-11-04

**Authors:** Ne N. Wu, Yingmei Zhang, Jun Ren

**Affiliations:** ^1^Department of Cardiology, Zhongshan Hospital, Fudan University, China; ^2^Shanghai Institute of Cardiovascular Diseases, Shanghai 200032, China; ^3^Center for Cardiovascular Research and Alternative Medicine, University of Wyoming College of Health Sciences, Laramie, WY 82071, USA

## Abstract

Biological aging is an inevitable and independent risk factor for a wide array of chronic diseases including cardiovascular and metabolic diseases. Ample evidence has established a pivotal role for interrupted mitochondrial homeostasis in the onset and development of aging-related cardiovascular anomalies. A number of culprit factors have been suggested in aging-associated mitochondrial anomalies including oxidative stress, lipid toxicity, telomere shortening, metabolic disturbance, and DNA damage, with recent findings revealing a likely role for compromised mitochondrial dynamics and mitochondrial quality control machinery such as autophagy. Mitochondria undergo consistent fusion and fission, which are crucial for mitochondrial homeostasis and energy adaptation. Autophagy, in particular, mitochondria-selective autophagy, namely, mitophagy, refers to a highly conservative cellular process to degrade and clear long-lived or damaged cellular organelles including mitochondria, the function of which gradually deteriorates with increased age. Mitochondrial homeostasis could be achieved through a cascade of independent but closely related processes including fusion, fission, mitophagy, and mitochondrial biogenesis. With improved health care and increased human longevity, the ever-rising aging society has imposed a high cardiovascular disease prevalence. It is thus imperative to understand the role of mitochondrial homeostasis in the regulation of lifespan and healthspan. Targeting mitochondrial homeostasis should offer promising novel therapeutic strategies against aging-related complications, particularly cardiovascular diseases.

## 1. Background

Biological aging is associated with a gradual decline in the organismal reproductive and regenerative capacity although a dramatic individual variation exists in the rate of decline [1]. To this end, chronological age may not be the best and authentic index for the prediction of individual health status. Healthspan offers an overlapping albeit distinct aging phenotype and is considered the ultimate goal for the elderly [2–4]. Maneuvers targeting the biological aging process are expected to ameliorate aging-related complications and improve well-being in the elderly [5]. According to the 2019 Statistical Update from the America Heart Association, cardiovascular disease (CVD) remains the leading cause of disability and death (17.6 million mortality in 2016, a 14.5% rise from 2006) with an expense predicted at $1.1 trillion in 2035 in the United States [6]. From a physiological perspective, intrinsic functional decline over time is expected to render the cardiovascular system more vulnerable to pathological stresses, resulting in a disproportionate prevalence of cardiovascular diseases with advanced age [7].

**Figure 1:**
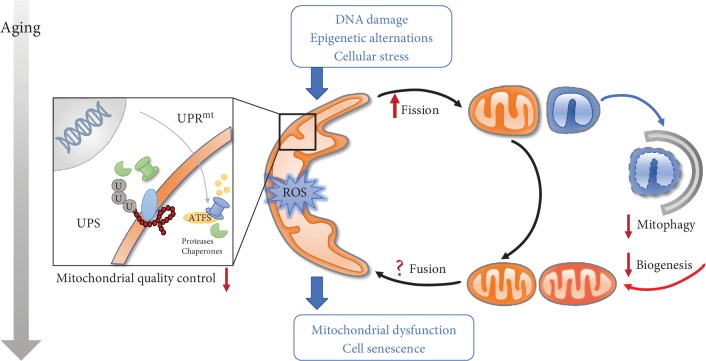
Unbalanced mitochondrial dynamics and turnover during aging. Mitochondrial homeostasis is maintained by a series of protective mechanisms. There is an overall decline of mitochondrial function with aging. A mitochondrial quality control system fails to repair mitochondrial defects. A mitochondrial network is progressively compromised due to loss of balanced mitochondrial fission and fusion. Inefficient mitophagy finally leads to buildup of dysfunctional mitochondria. UPS: ubiquitin-proteasome system; UPRmt: mitochondrial unfolded protein response.

Cardiovascular aging refers to age-related deterioration of cardiovascular function and is manifested as the loss of myocardial contractile capacity including increased left ventricular (LV) wall thickness and chamber size, prolonged diastole [8, 9], as well as loss of compliance in LV wall and coronary vasculature, arterial stiffness, and endothelial dysfunction [8, 10–12]. Up to date, a number of theories have been postulated for the pathogenesis of aging-related cardiovascular dysfunction including oxidative stress, DNA damage, telomere shortening, genomic instability, epigenetic and metabolic disarray, inflammation, apoptosis, lipotoxicity, and mitochondrial injury [13–15], among which mitochondrial injury has received close attention over the past decades. Mitochondria are double-membraned organelles found in eukaryotic cells capable of producing adenosine triphosphate (ATP) utilized for nearly all of the biological processes. Mitochondria are entangled in multitasks beyond energy production, such as susceptibility to cell stress and cell fate determination [16]. With aging, mitochondria usually display a gradual although dramatic decline in abundance, integrity, dynamics, purging, and bioenergetic efficiency [17]. Defects in mitochondria are commonly reflected as accumulated mtDNA mutation, impaired metabolism, inflammatory responses, deformation (swelling and shrinkage), and cell senescence [17–21], thus contributing to a myriad of aging-related disease phenotypes, such as neurodegenerative diseases, metabolic disorders, cancer, and cardiovascular diseases [22–26]. Mitochondria make up nearly 1/3 of cellular volume and are vital for all cellular processes including metabolism, energy, intracellular Ca^2+^ handling, and redox homeostasis [27]. Disturbance in mitochondrial homeostasis under pathological stress leads to reactive oxygen species (ROS) production and energetic insufficiency, which further disrupt mitochondrial and cellular homeostasis into a vicious cycle [28]. The precise control of mitochondrial homeostasis through a well-orchestrated yet complex network of antioxidants, DNA repair, and mitochondrial quality control systems helps to maintain a pool of healthy and functional mitochondria [29]. Furthermore, mitochondria are highly dynamic and constantly undergo morphological changes between fission (division) and fusion in response to various metabolic and environmental cues. A fusion process assists to homogenize the contents of damaged mitochondria resulting in mitochondrial elongation. Fission, on the other hand, leads to mitochondrial fragmentation and promotes clearance of damaged mitochondria through a form of selective autophagy-mitophagy [30]. Excessive or untimely fission or fusion may be detrimental to mitochondrial quality and mitochondrial homeostasis (Figure 1). The identification and manipulation of molecules involved in mitochondrial dynamics such as dynamin-related protein 1 (DRP1) and Fis1 have greatly added breadth to our understanding for mitochondria in biological particularly cardiovascular aging [31]. Intriguingly, an organism seems to be much more tolerant to poor mitochondrial efficiency than one would expect, and certain mitochondria-related deviations and modulations are proven to benefit healthspan [32]. In this minireview, we will highlight key components of mitochondrial fission-fusion, mitophagy, and mitochondrial homeostasis and their roles in the biology of aging and aging-related cardiovascular diseases. We used the key terms of “aging,” “mitochondria,” “quality control,” and “mitophagy” as the key terms to search PubMed over the last 5 years.

## 2. Overview of Mitochondrial Fission-Fusion

Fission-fusion processes play a vital role in the dynamic regulation of mitochondria. In mammals, several dynamin-related GTPases participate in the mitochondrial fusion process: the mitofusins (Mfn1 and Mfn2) for the fusion of the outer mitochondrial membrane (OMM) and optic atrophy 1 (OPA1) for the inner membrane fusion [33]. Constitutive processing of OPA1 at proteolytic cleavage sites generates two isoforms of OPA1 [34]: long-OPA1 (L-OPA1) and short-OPA1 (S-OPA1), which cooperatively modulate the mitochondrial fusion state. Several proteases such as OMA1, YME1L (an i-AAA protease), and AFG3L2 (an m-AAA protease) regulate OPA1 variants [35]. More recent findings suggested that L-OPA1 is sufficient for mitochondrial fusion through its binding with cardiolipin (CL) on the opposite membrane and homotypic interaction of OPA1 mediates IMM tethering and formation of cristae [36]. Stress-induced rapid proteolytic cleavage of OPA1 into short forms participates in mitochondrial fragmentation [37]. These mitochondrial fusion proteins may be ubiquitination by several E3 ligases such as Parkin and MARCH5/MITOL and then get degraded by proteases, leading to decreased mitochondrial fusion and autophagic degradation of mitochondrial organelles [38, 39]. It was reported that overexpression of appoptosin, a mitochondrial carrier protein located on IMM, compromised the interaction between Mfn1 and Mfn2, resulting in mitochondrial fragmentation [40].

For mitochondrial fission, dynamin-related protein 1 (Drp1) plays a paramount role. Drp1 adaptors such as mitochondrial fission 1 (Fis1), mitochondrial dynamics proteins of 49 and 51 kDa (MiD49/51), and mitochondrial fusion factor (Mff) cooperatively or independently form fission sites where Drp1 gathers to assemble the higher-ordered spiral complexes that constrict mitochondria for division [41]. Drp1 may constantly oligomerize on mitochondria although such process does not sufficiently trigger fission. The presence of certain fission factors, such as actin filaments, promotes the progressive maturation of Drp1 oligomers and uneven division due to unequal membrane potential [42]. Drp1 is also regulated by posttranslational modifications and metabolic signals [43]. Mdivi-1 is known to inhibit Drp1-dependent fission, while recent studies indicated that Mdivi-1 may not be a specific inhibitor of Drp1 and can reversibly inhibit mitochondrial complex I [44]. The presence of mitophagy and mitochondrial division in the Drp1-defective cells prompted the recognition of several novel mediators of fission, such as Tmem135 [45]. For instance, phagophores could emerge and elongate on a budded portion of mitochondria. Mitochondria are divided when the phagophore is closed without Atg5/Agt3 [46]. These data suggest the possible presence of atypical mitochondrial fission. A work reported by Fonseca and coworkers reconfirmed the crucial role of Drp1 in fission while dynamins (DNM1-3) are dispensable [47].

## 3. Mitochondrial Dynamics and Aging

Historically, mitochondrial dynamics resides in bioenergetic adaption to favor integrated or fragmented morphology of organelles. Mitochondrial dynamics was at one time difficult to capture in cultured cardiomyocytes, where proteins essential to these processes are abundant [48]. With the advancement of modern imaging technology, scientists have captured robust mitochondrial fusion and fission in healthy cardiomyocytes promptly after isolation. It has been suggested that well-functioning transition through fusion and fission is crucial for normal cardiomyocytes. Emerging evidence suggests that disrupted mitochondrial dynamics negatively impacts mitochondrial function and myocardial survival, resulting in the aging-induced buildup of dysfunctional mitochondria [49].  Table 1 summarizes evidence of mitochondrial dynamics in longevity and cardiovascular diseases.

Given the constant high-energy demand for cardiac contractility, a broadly connected mitochondrial network is essential for cardiomyocytes [50]. Studies from C. elegans revealed the role of increased mitochondrial fusion as a potent avenue to reconstitute a productive mitochondrial network [51]. Wai and associates found that ablation of Yme1L in a mouse heart activated OMA1 and OPA1 proteolysis, which induced mitochondrial fragmentation, dilated cardiomyopathy, and heart failure [52]. Furthermore, mitochondrial fusion could possibly preserve mitochondrial mass against pathological insults such as aging [53]. Mitochondrial fusion under the regulation by CAND-1 and SCF^LIN-23^ is responsible for increased elongation of mitochondria and is required in longevity signaling such as insulin/IGF-1 signaling inactivation, physical exertion, caloric restriction, TOR (LET-363) inactivation, activation of sirtuin (SIR-2.1), and AMPK [54]. All these long-lived animal models presented an elongated mitochondrial network and generated less mitochondrial ROS [54]. The extended lifespan was significantly shortened upon the treatment with *eat-3* RNAi to interrupt mitochondrial fusion [54]. Consistent with this notion, Byrne and coworkers reported that lack of fusion (*eat-3*, *foz-1*) or fission proteins (*drp-1*) in C. elegans impacted movement and neuronal function and significantly reduced median lifespan without affecting the maximal lifespan. Moreover, interruption of fusion displayed a potent impact on median lifespan (12, 13, and 15.6 days for *eat-3* mutants, *foz-1* mutants, and *drp-1* mutants, respectively) in comparison with the wild type (20 days) [55]. Despite the fact that mitochondrial fusion is required for longevity, a fine balance between fusion and fission is vital for pathological changes including cardiovascular diseases. Defects in one process could be temporarily alleviated by a concomitant suppression of other processes in a compensatory manner [56]. Concomitant disruption of mitochondrial fission diminishes mitochondrial fragment and improves mitochondrial function triggered by lessened fusion, indicating an essential role for the maintenance fission-fusion balanced in the face of disrupted mitochondrial fusion under pathological stresses [55, 57].

In physiological conditions, Drp1-mediated fission may set a “strict” threshold for mitophagy and protect healthy mitochondria from “unchecked” mitophagy. Coronado and colleagues suggested that physiological fission is required for cardiac adaptation in response to normal energy stress, such as exercise [58]. It is also reported that Drp1 deletion in normal conditions with low levels of Parkin provoked hypermitophagy through upregulating Parkin and contributed to mitochondrial depletion and lethal cardiomyopathy [59]. Paradigms have held that segregation of depolarized portions of mitochondria via fission facilitates mitophagy to remove damaged or long-lived mitochondria, preserving mitochondrial homeostasis under stresses. Shirakabe and colleagues proposed that upregulation of fission and autophagy in acute settings may protect the mitochondria and heart from pressure overload, while suppression of Drp1-dependent mitochondrial autophagy could be responsible for cardiac pathology. They found that nonselective autophagy (within 24 hours), Drp-1 mediated fission [2-3 days after transverse aortic constriction (TAC)], and mitochondrial autophagy (3-5 days after TAC) were transiently activated in mouse hearts after TAC. Interestingly, autophagy and fission were significantly suppressed below physiological levels during the second phase (after 5-7 days for autophagy and after 14 days for Drp1). Heart failure developed after mitochondrial fragmentation, whereas Drp1 may return to normal levels along with suppressed autophagy. Haploinsufficiency of Drp1 abolished mitophagy and exacerbated heart failure, while protective mitophagy elicited by Tat-Beclin was abrogated by removal of Drp1 [60].

However, the regulation of mitochondrial fission declines during aging. This notion may be echoed by diminished mitophagy in aging, in concert with mitochondrial fission. D'amico and associates showed that RNA-binding protein Pumilio2 (PUM2), a translation repressor, decreased with age and downregulated translation of Mff, which hampered mitochondrial fission and mitophagy and promoted age-related mitochondrial dysfunction [61]. Moderately sustained stimulation of fission could protect against aging by unidentified mechanism(s). Rana and colleagues reported that short-term induction of Drp1 in midlife, but not earlier, extended both the lifespan and healthspan of Drosophila melanogaster. Midlife induction of Drp1 resulted in a decrease of p62 accumulation to mitochondria, while lack of Atg1 in midlife eliminated the benefits of Drp1, indicating that mitophagy may play a role in the beneficial effects of Drp1 [62].

A shift was noted from fusion to fission under multiple pathological conditions, indicating a possible role for mitochondrial fission in cardiovascular diseases [63]. Several studies suggested that unchecked fission and mitophagy are, at least in part, responsible for the development of cardiac aging, while interventions that limit excessive mitochondrial fission were suggested to offer cardioprotective effects in such pathological processes. Mdivi-1 treatment given prior to ischemia significantly improved cardiac function and reduced infarction size and arrhythmia [64]. Doxorubicin-treated H9c2 myocytes exhibited mitochondrial fragmentation and accelerated mitophagy, while RNA-mediated knockdown of Drp1 decreased cell death and attenuated cardiac damage [65]. It was reported that melatonin could prevent myopathy by inhibiting diabetes-induced activation of Drp-1 and fission through a Sirt1-PCG1*α*-dependent manner [66]. A novel regulator of mitochondrial fission Tmem135 has also been noted in cardiovascular diseases. It was reported that overexpression of Tmem135 induced mitochondrial fragmentation and exaggerated collagen accumulation and hypertrophy, which exhibited similar gene expression patterns and disease phenotypes to those found in aging [67]. Furthermore, mitochondrial fission may induce cellular death in extreme conditions. Excessive mitochondrial fission is induced in cardiac ischemic injury and leads to a higher susceptibility to mitochondrial permeability transition pore (mPTP) opening and apoptosis during reperfusion phase [68]. In addition, fission may indirectly attenuate the aging process. Increased mitochondrial fission is also associated with high proliferation in some cancer cells and with low differentiation in stem cells [69]. Drp1-mediated mitochondrial fission is required to remove apoptotic cells by phagocytes, resulting in alleviation of postapoptotic necrosis and inflammation [70]. Whether fission-mediated anti-inflammation benefits cardiac aging has not been well understood. Despite ample evidence consolidating the benefit of inhibiting Drp1-mediated fission in pathological conditions, its effectiveness may be model-dependent. Using a large animal (pig) model of acute myocardial infarction, Mdivi-1 treatment given at the onset of reperfusion failed to preserve LV function or reduce myocardial infarction size. Furthermore, these authors revealed little change in fission after Mdivi-1 treatment, suggesting the necessity of more specific Drp1 inhibitors [71].

## 4. Mitochondrial Quality Control

More than 1000 proteins encoded by nuclear genes reside in mitochondria to integrate the network that governs mitochondrial biogenesis, morphology, and function [72]. Proper folding, translocation, and assembly of proteins are fundamental to mitochondrial homeostasis. In addition, mitochondria constantly produce energy in response to metabolic alternations and environmental cues at the cost of erosion by metabolic byproducts, such as ROS [73]. Failure to achieve structural integrity leads to accumulation of protein aggregates and dysfunctional organelles with aging [4]. To counter these culprits, several mechanisms may emerge as follows: (1) Dedicated chaperones and proteases degrade aberrant proteins within the matrix and intermembrane space (IMS) [74]. (2) A cytosolic ubiquitin-proteasome system (UPS) ubiquitinated proteins for subsequent destruction by the 26S proteasome in a p97-dependent manner [75]. (3) The mitochondrial unfolded protein response (UPRmt) relays stress signals retrograde to the nucleus and transcriptionally upregulates mitochondrial chaperones and proteases, such as ClpP (protease) and mtHsp60 (chaperone) to promote folding and degradation capacity [76]. (4) Mitochondria-derived vesicles (MDVs) transport damaged portions to the late endosome/lysosome and even to neighboring cells for degradation in hopes of preserving the undamaged part [77]. (5) Mitochondrial dynamics, biogenesis, and clearance of damaged mitochondria by mitophagy cooperate with each other to conserve mitochondrial fitness and cellular homeostasis [78]. Mitochondria also support quality control systems in extramitochondrial compartments through interconnected processes. It has been found in yeast that cytosolic aggregation-prone proteins are imported into mitochondria for degradation with the help of a chaperone protein Hsp104 to dissociate aggregates [79]. Mitochondria may act as a transient disposal unit in cells, where the wastes is sorted and destroyed [80].

High-energy stress imposes mitochondria more prone to injury. Such high-energy demand tissues such as the myocardium are also more sensitive to mitochondrial dysregulation, where slight tinkering is not enough. Thus, selective degradation of mitochondria is imperative here to recycle useful constituents and then restore the fidelity of mitochondria. Mitophagy specifically recognizes and removes defective mitochondrial units that would otherwise be detrimental to organismal health. Given the high-energy demand of cardiomyocytes, here, we mainly talk about the role of mitophagy in cardiac aging rather than other cellular stress responses.

### 4.1. Molecular Mechanisms of Mitophagy and Biogenesis

Mitophagy is one kind of selective autophagy, which targets long-lived or damaged mitochondria to degradation. In mammals, the molecular mechanism of mitophagy was first elucidated in mitochondrial clearance during erythropoiesis, which requires Nip3-like protein X (NIX/BNIP3L) [81]. The transmembrane mitophagy receptors, such as NIX, BNIP3, and FUNDC1, harbor a LC3-interacting region (LIR) motif, allowing formation of a bridge between ligands on the OMM with LC3/GABARAP, the mammalian autophagy-related 8 (Atg8) homologs attached to the autophagosomes [82, 83]. Receptor-mediated mitophagy is generally activated in response to cellular differentiation cues (NIX) [84] and some acute stresses, such as hypoxia (BNIP3, NIX and FUNDC1), nutrient stress (BNIP3), and ischemia-reperfusion [85]. For instance, FUNDC1 promotes mitophagy in response to hypoxia upon dephosphorylation by PGAM5, while casein kinase 2 (CK2) reverses the FUNDC1 activation process by phosphorylating FUNDC1 at Ser^13^ [86]. BcL2L1/Bcl-xL suppresses mitophagy through binding to PGAM5 and preventing the dephosphorylation of FUNDC1 [87].

PINK1 (PTEN-induced putative kinase protein 1), a more well-known mitophagy mediator, accumulates on the depolarized mitochondria, where PINK1 proteolysis is compromised, and phosphorylates the E3 ubiquitin ligase Parkin. Parkin tags proteins embedded in the OMM with PINK1-generated phosphoubiquitin, which then become substrates of PINK1, feeding back to amplify autophagic signals [88]. Cytosolic autophagy receptors, such as optineurin (OPTN), nuclear dot protein 52 kDa (NDP52), and (to a lesser degree) TAXBP1, but not p62, bind ubiquitin chains on the targeted mitochondria to processed LC3/GABARAP [89], whereas more recent studies suggested that LC3-II is not mandatory for autophagosome formation in PINK1-Parkin-mediated mitophagy or starvation-induced autophagy [90]. Consistent with this notion, it was reported that OPTN and NDP52 induce local recruitment and activation of autophagy factors like ULK1, DFCP1 (double FYVE domain-containing protein 1), and WIPI1 (WD repeat domain phosphoinositide-interacting protein 1) proximal to mitochondria, which likely contributes to autophagosomal formation de novo on injured mitochondria or lysosomal targeting to damaged mitochondria.

Alternatively, mitophagy may be regulated differently from how general autophagy is regulated such as in the absence of Atg5 or Atg7 (alternative autophagy) and thus cannot be evaluated with conventional markers such as LC3-II. The ATG5/ATG7-independent autophagy, which depends on the ULK (Unc-51-like kinase) and Beclin1 complexes, plays a prominent role in mitophagy induction [91]. Cardiolipin (CL) on the OMM could also bind to another Atg8 human ortholog LC3B and induce mitophagy [92]. Endoplasmic reticulum (ER) and mitochondria form tight functional contacts that regulate several cellular processes. PINK1 and Beclin1 translocate to specific regions of ER-mitochondria contact, namely, mitochondria-associated membranes (MAM) and promote the formation of autophagosomes [93]. Autophagosome could also form from other sources, such as Golgi vesicles in a GTPase Rab9-dependent manner [94]. It was reported that mitochondria may be sequestrated into the early Rab5-positive endosomes through the ESCRT machinery before being delivered to lysosomes for degradation [95]. Intriguingly, the lost or unuse of Parkin does not block mitophagy [96], but that does not imply Parkin has no part to play because Parkin dramatically increases mitophagy through ubiquitylating many proteins on OMM directly or indirectly involved in mitophagy, including Mfn1/Mfn2, PGC-1*α*, and NIX [97, 98].

To rejuvenate mitochondrial mass, mitochondrial biogenesis is also required to provide new and healthy “new blood” to the mitochondrial pools [99]. Mitochondria are semiautonomous organelles. Mitochondrial DNA (mtDNA) encodes 13 essential subunits of the oxidative phosphorylation (OXPHOS) system, with their levels depending on spatiotemporal coordination with nucleus genes [100]. Thus, identification of a single particular regulator of mitochondrial biogenesis is difficult. In general, peroxisome proliferator-activated receptor gamma coactivator 1-*α* (PGC-1*α*) is thought to act as a central hub in fine-tuned crosstalk between mitophagy and mitochondrial biogenesis. PGC-1*α* may interact with transcription factors, such as peroxisome proliferator-activated receptor (PPAR*β*), nuclear respiratory factor (NRF), and estrogen-related receptors (ERR) to orchestrate the overlapping gene expression in mitochondrial biogenesis. PGC-1*α* can also promote mitochondrial fusion and inhibit mitochondrial fission through regulating Mfn2 and Drp1 [101].

### 4.2. Role of Mitophagy in Cardiac Aging

Defective segments of mitochondria are segregated from the rest of the mitochondrial network through fission for elimination by mitophagy. Fragmented mitochondria and decreased baseline of mitophagy have been noted in aging hearts [102]. Several proteins involved in mitochondrial turnover such as PINK1 and PGC-1*α* tend to decrease in old animals. These data indicated a decline in the function and regulation of mitophagy during aging [103]. Recent studies suggested that aging-related mtDNA mutations may disrupt the receptor- (NIX and FUNDC1) mediated mitophagy in the differentiation process in adult cardiac progenitor cells (CPCs), which resulted in sustained fission and less functional fragmented mitochondria [104]. Therefore, some activators of mitophagy have been used in aging models and showed some beneficial effects. For instance, urolithin A has been widely reported to extend lifespan in C. elegans and improve physical exercise capacity in rodents through upregulating mitophagy [105]. However, why and how mitophagy declines during aging have not been well defined. Several hypotheses were speculated thus far. Rizza and colleagues reported S-nitrosoglutathione reductase (GSNOR/ADH5), a protein denitrosylase that regulates S-nitrosylation, was downregulated with aging in mice and humans [106]. Accumulation of S-nitrosylation severely impaired mitophagy, rather than autophagy, leading to hyperactivated mitochondrial fission by targeting Drp-1 in GSNOR^−/−^ mice and cells [106]. It is noteworthy that expression of ADH5 is sustained in long-lived individuals, indicating the potential of ADH5 and S-nitrosylation as targets in the aging process through selectively modulating mitophagy [107]. Manzella and associates observed that ROS produced by mitochondrial enzyme monoamine oxidase-A (MAO-A) resulted in cytosolic accumulation of p53, one of the classic markers for cellular senescence. p53 further suppressed Parkin and therefore inhibited mitophagy, leading to mitochondrial dysfunction, which suggested a possible mechanism of MAO-A-induced oxidative stress in an age-related process [108]. Certain mitophagy proteins are directly or indirectly involved in the aging process. Parkin enhances transmission and replication of mtDNA in the presence of TFAM (mitochondrial transcription factor A) in proliferating cells [109]. TFAM is known to promote mtDNA through packaging mtDNA into mitochondrial nucleoids [110]. Chimienti and coworkers examined TFAM binding to mtDNA in aged (28 months) and extremely aged rats (32 months) and revealed a significant drop in TFAM binding in the extremely aged rats [111].

In essence, mitophagy is considered a self-defense and garbage removal process that maintains mitochondrial homeostasis and cellular health, in the face of pathological stimuli. Knuppertz and coworkers found that a PaSOD3 (P. anserine mitochondrial superoxide dismutase) deletion strain of ascomycete Podospora anserine surprisingly displayed similar lifespan as wild type, though superoxide accumulated and respiratory chain was impaired. They noted that mitophagy was predominantly induced by superoxide in the old PaSOD3 deletion strain. To verify whether autophagy is permissive to maintain the deficient strain healthy, they concomitantly ablated PaATG1 (ULK1 in mammals) and PaSOD3 and found a significant decrease in lifespan. They also elaborated the double sides of autophagy and mitophagy—with mild stress triggering protective level of autophagy and severe stress prompting excessive mitophagy, therefore provoking predeath pathways and accelerating aging [112], whereas the optimum state of mitophagy remains controversial and the impact of mitophagy may differ depending on pathological conditions. Both positive and negative effects of mitophagy in ischemia-reperfusion (IR) have been reported among various organs including the hearts, brains, and kidneys. Relatively, more evidence supported the positive side of mitophagy in cardiac IR. Zhou and coworkers found that NR4A1 was markedly increased following IR injury, accompanied with facilitated Mff-mediated fission and suppressed FUNDC1 mitophagy through a CK2*α*-mediated mechanism, leading to mitochondrial damage and microvascular collapse [113]. These authors suggested that increased Ripk3 may induce mitochondria-mediated apoptosis in cardiac IR via suppressing mitophagy, while Ripk3 deficiency reduced apoptosis and protected mitochondrial against IR damage in a mitophagy-dependent manner [114]. It was reported that melatonin could prevent cardiac IR injury through activating AMPK-OPA1-mediated fusion and mitophagy [115]. Mammalian STE20-like kinase 1 (Mst1) significantly increased in a reperfused heart, which suppressed FUDC1-mediated mitophagy and induced proapoptosis signals. Mst1 knockout mice could reverse FUNDC1 expression and markedly reduced the myocardial infarction (MI) size [116]. Besides acute activation of protective mitophagy mentioned above, it should be noted that mitophagy also plays a role in chronic cardiac diseases. A recent study reconfirmed that mitophagy is crucial for mitochondrial homeostasis and cellular health in mice in response to a high-fat diet. Inhibition of mitophagy enhanced mitochondrial defect and lipid accumulation and thus deteriorated diabetic cardiomyopathy [117]. Moreover, Mst1 possibly participated in the development of diabetic cardiomyopathy through inhibiting Sirt3-related mitophagy [118]. It was demonstrated that simvastatin may prevent angiotensin II-induced heart failure through promoting autophagy and mitophagy and increasing lipid droplets in cardiomyocytes, contributing to the maintenance of mitochondrial quality and function [119]. Mitophagy has also been reported to combat against drug-induced cardiotoxicity. A mouse model of doxorubicin-induced cardiotoxicity showed decreased Rubicon expression and mitophagy 16 hours after intraperitoneal injection. Therefore, targeting doxorubicin-induced inhibition of mitophagy, autophagy flux, and mitochondrial dynamics may represent a novel avenue for doxorubicin cardiomyopathy [120].

On the contrary, mitophagy may also provide unfavorable effects on the heart. Feng and associates reported GPER (G protein-coupled estrogen receptor 1) protects a mouse heart from IR injury at the onset reperfusion through downregulating mitophagy [121]. Advanced glycation end products (AGEs) significantly increased the number of senescent cells in neonatal rat cardiomyocytes, coinciding with activation of PINK1/Parkin-mediated mitophagy [122]. These different effects shown above may result from the different levels, types, and duration of mitophagy; the method and time points of treatments; and the intrinsic differences of different models in these studies. In addition, two phases of IR exhibit different features, including the basal mitophagy, which we will talk about later. Despite much work has been done to clarify why autophagy could be harmful, more studies are still needed to clarify the underlying mechanism of mitophagy-induced damage. Zhou and coworkers recently reported that increased mitochondrial permeability is attributed to switching autophagy into a harmful force in mammals. Serum/glucocorticoid regulated kinase-1 (SGK-1) is required for degradation of mPTP component VDAC1 in both C. elegans and mammalian cells [123]. They found that C. elegans lacking SGK-1 presented overly activated mTORC2-induced autophagy and short lifespan [123].

Dozens of species have depicted a unique protective role of mitophagy in aging and cardiovascular diseases, an effect consistent with suppressed mitophagy in multiple pathways. The baseline of mitophagy in different cardiac diseases may help understand the complex effects of mitophagy. The presence of a switch from AMPK*α*2 to AMPK*α*1 in failing hearts has been well documented, leading to a decrease of AMPK*α*2-mediated mitophagy and development of heart failure [124]. In another independent study, upregulated CK2*α* following acute cardiac IR injury was found to suppress FUNDC1-mediated mitophagy, leading to infarct area expansion and cardiac dysfunction [85]. Furthermore, ischemia activated FUNDC1-mediated mitophagy while reperfusion suppressed mitophagy possibly through activating Ripk3 [114]. Not surprisingly, interventions that restored mitophagy to normal levels, but not above normal levels, in these conditions should help to maintain mitochondrial homeostasis and cellular function. For instance, hypoxic precondition was recognized to suppress the activation of platelets and I/R injury in the heart through increasing FUNDC1-mediated mitophagy [125]. Exercise was reported to restore autophagic flux and mitochondrial oxidative capacity after myocardial infarction [126].  Table 1 summarizes evidence from a cadre of mitophagy in longevity and cardiovascular diseases.

Generation of new mitochondria through mitochondrial biogenesis plays a vital role in populating mitochondrial pool with adequate numbers and mass [127]. There has been some evidence to suggest the benefits of mitochondrial biogenesis during aging. PGC-1*α* overexpression improves lysosomal capacity and autophagy but reduces aging-associated mitophagy and ameliorates a mitochondrial defect [128, 129]. Several studies tried to associate the loss of PCG-1*α* with aging-related diseases, while its effects in cardiovascular diseases are less known. PCG-1*α*^+/-^ mice fed a high-fat diet for 4 months presented age-related macular degeneration- (AMD-) like abnormalities in retinal pigment epithelium (RPE) as well as decreased mitochondrial activity and increased ROS [130]. Muscle-specific upregulation and downregulation of PCG-1*α*, respectively, alleviated and exacerbated age-related muscle loss in mice. PCG-1*α* is also required for the muscle benefits of endurance exercise training [131].

## 5. Mitochondrial Adaptation and Metabolic Signals

Mitochondrial adaptation is partially established by metabolic signal molecules and epigenetic mechanisms that orchestrate gene expression underlying the generation and the removal of mitochondria. Several nutritional sensors, such as mTOR (mechanistic target of rapamycin), AMPK (AMP-activated protein kinase), and sirtuins, are involved in the processes that link environmental and intracellular stimuli to mitochondrial morphology and turnover. Generally, mTORC1 is recognized as a negative regulator of autophagy, while AMPK and SIRT1 facilitate autophagy and induce PGC-1*α*-mediated biogenesis [132]. AMPK extended lifespan through reviving the youthful mitochondrial homeostasis via both fusion and fission [133]. A novel stress-induced protein, sestrin2, declines with aging, which hampered the activation of AMPK, leading to reduced substrate metabolism and increased sensitivity to ischemia injury [134]. mTORC1 may promote fission in high caloric intake conditions through upregulating the translation of mitochondrial fission process 1 (MTFP1), which is coupled with the activation and recruitment of Drp1 [135]. Lang and colleagues detected increased autophagy flux and significantly decreased Parkin-induced mitophagy under stress conditions in HEK293 cells with stable expression of Sirt4 [136]. Sirt4 tilted the mitochondrial dynamic balance towards fusion and counteracted fission as well as mitophagy possibly via interacting with L-OPA1 [136]. Sirt3 has been intensively discussed given its protective role in cardiac IR injury through multiple mechanisms. Sirt3 may either activate or inhibit autophagy, which may be used to maintain an optimal range of autophagy in different phases of IR [137]. Chen and colleagues reported that sustained exercise improved Sirt1, AMPK*α*1, and PCG-1*α* and attenuated aging-associated cardiac inflammation in D-galactose-induced aging mice [138]. Not surprisingly, the sirtuin cofactor NAD+ activates Sirt1 and a range of transcription factors that may decelerate aging, making it an emerging focus in the field of aging. Mitochondria control the concentration of NAD+ in cellular [139]. Katsyuba and coworkers showed that *α*-amino-*β*-carboxymuconate-*ε*-semialdehyde decarboxylase (ACMSD) limits de novo NAD+ biosynthetic pathway across species, including C. elegans, rats, and human [140]. Inhibition of ACMSD boosted NAD+ synthesis, prolonged lifespan in worms, and prompted a more extensive and interconnected mitochondrial network in C. elegans [140]. On the contrary, ablation of ACMSD was reported to enhance mitochondrial functions in human hepatocytes, indicating the complexity of NAD+ biosynthesis in organ function [140]. eNAMPT, one of the rate-limiting enzymes in the NAD+ biosynthetic pathway, declines with age in mammals, including human. A more recent study demonstrated that genetic supplementation or extracellular vesicle-mediated supplementation of eNAMPT could extend lifespan in mice, which prompted to the scrutiny of mitochondria in the longevity-defining processes outside mitochondria itself [141]. In C. elegans, SKN-1 (the nematode NRF) senses metabolic signaling and initiates a retrograde response towards the mitophagy-related DCT-1 (NIX/BNIP3L homolog) [142]. It was reported that autophagy and lysosomal biogenesis-related gene transcription factor EB (TFEB) exhibit parallel changes with that of PGC-1*α*. The putative nutrient-sensing regulator GCN5L1 (general control of amino acid synthesis 5-like 1) may restrain both mitochondrial biogenesis and degradation through direct transcriptional suppression of TFEB [143].

### 5.1. From Middle Age to Old Ages

Mitochondrial adaptation could be either beneficial or detrimental throughout life; the effects of mitochondrial dynamics and mitophagy largely depend on the type, level, and duration of stresses and the overall condition of individuals. Here, we are trying to depict a simplified picture about how mitochondria adapt throughout the entire life, which may imply some possible strategies to prolong healthspan. There is a transition from neonatal glycolysis to mitochondrial oxidative mechanism that mainly utilizes fatty acids in adulthood. Parkin-mediated mitophagy, through interaction with mitochondrial biogenesis, contributes to this metabolic remodeling by way of replacing old mitochondria with new ones that contain different enzymes and substrates [144]. Mild stress could make mitochondria more tolerant and adaptable. Senchuk and colleagues studied three C. elegans and mitochondrial mutants and found that increased ROS activated FOXO transcription factor DAF-16 and contributed to their longevity [145]. Moreover, a set of molecules released from mitochondria, such as NAD+, NADPH, ROS, iron-sulfur cluster, and Ca^2+^, has been determined as “second messengers” that affect the efficiency of some aging or antiaging processes throughout the lifetime [139]. However, both cellular homeostasis and mitochondrial functions are crucial, while mitochondria would sometimes sacrifice their own quality and integrity to accomplish its mission. Li and associates reported that the MOM protein FUNDC1 interacted with HSC70 to promote the unfolded protein response, but excessive accumulation of unfolded protein on the mitochondria impaired mitochondria and fatally evoked the pathways leading to cell senescence [146].

Accumulating observations have suggested a biphasic model of mitochondria wherein the metabolic rate is increased from youth to middle age and then drops again at older ages [147]. Rana and colleagues observed a decline of Drp-1 in the midlife of Drosophila, which may contribute to the more elongated mitochondria and then a decline in mitophagy [62]. On the one side, a midlife shift toward mitochondria fusion in response to relatively mild stress seemed to benefit temporarily but turned out to be potential threats later in life since reduced fission and accompanying deficiency of mitophagy resulted in the accumulation of dysfunctional mitochondria [62]. Byrne and coworkers performed several behavioral assays in C. elegans and revealed that disruption of mitochondrial fission progressively reduced animal movement in older drp-1 mutants, but displayed fewer defects in early adulthood than that of fusion mutants, which provided further support to the notion that fission is crucial in late life in response to severe stressors [55]. On the other hand, midlife promotion of mitochondrial capacity may link to the chronic cell senescence [148]. Cell senescence is another hallmark of aging, a stage of terminal cell cycle arrest characterized by high metabolic activity and hypersecretion of proinflammatory and prooxidant signals, termed senescence-associated secretory phenotype (SASP), which requires massive activation of mitochondria and interacts with senescent-associated mitochondrial dysfunction (SAMD) [149]. Some observations indirectly support the postulation that mitochondria excessively fused for abundant energy at the cost of reduced fission and mitochondrial quality: early intervention of caloric restriction is sufficient to expand lifespan and preemptively reduce age-related diseases in diverse species [150], while higher energy expenditure increases the risk of premature death in human life [151].

From the “struggling” middle age to the “difficult” old age, resources are exhausted, where cellular stress responses are no longer robust. Mitochondrial defects progressively accumulate and ultimately become unrepairable by a mitochondrial quality control system. There is a progressive loss of mitochondrial network connectivity and a reduction in mitochondrial mass in aging cells of C. elegans [133]. The overt decline of mitochondria depresses ATP-linked respiration and exacerbates superoxide generation, which results in a compensatory higher oxygen consumption rate (OCR) to meet energetic requirements. Under certain pathological stress, mitochondria could switch from the key player in cell adaptive survival to the final executor of cell death through Bcl-2 family interaction-mediated proton leak and release of proapoptotic factors such as cytochrome C [152]. Recent studies in cancer cells revealed mitochondria-modulated apoptotic protein expression [153]. Likewise, mitochondrial dynamics and mitophagy could conversely act as maladaptive processes that amplify mitochondrial damage and apoptotic signaling [154]. Autosis, a form of cell death triggered by high levels of autophagy in response to stimulus-like pharmacological treatment, starvation, and ischemia, is mediated by the Na^+^-K^+^-ATPase pump and featured by increased autophagosomes/autolysosomes [155]. Starvation-induced abrogation of PGC-1*α* leads to p53-mediated apoptosis, indicating a possible link to cell fate determination [156]. Selective organelle clearance and cell death are not distinct processes; they perform hierarchically at the level of organelle or cell [157].

## 6. Conclusion and Future Perspectives

Mitochondria constitute a dynamic network interacting with other cellular compartments to orchestrate various physiological processes and cellular stress responses. Alterations in mitochondrial functions are proven to be a major contributor to aging and aging-related diseases, especially cardiovascular diseases. Mitochondrial dynamics, biogenesis, and turnover are essential for mitochondrial and cellular homeostasis. Aging is potentially malleable via metabolic and genetic interventions, such as caloric restriction and exercise [158]. Dietary supplements such as antioxidants offer limited benefit. Physiological and pharmacological inducers of mitophagy as well as modulators of mitochondrial dynamics improve mitochondrial function and healthspan in various model organisms [4]. Further progress in preserving and attenuating aging-induced pathologies should come from a better understanding of the causal mechanisms underlying aging itself and hopefully from targeting the mechanisms implicated in the regulation of mitochondrial homeostasis to counteract mitochondrial damage at an early stage. Perhaps, the way we fight aging lies in the way we treat with midlife.

## Figures and Tables

**Table 1:** Alterations in mitochondrial dynamics and turnover for aging and CVD.

Protein	Alteration	Age-related disease/phenotype	Organism/model	References
Mfn2	Reduced expression	Hyperproliferation of vascular smooth muscle cells	Rats or mice: hypertensive and atherosclerotic arteries	[159]
		Accelerated cardiac hypertrophy and cardiomyopathy	Mouse heart	[38, 160, 161]
Mfn1	Increased expression	Decreased glycolysis, increased oxygen consumption rate, and ATP levels	Old normal human fibroblasts	[162]
Opa1	Reduced expression	Accelerated heart failure	Heart from humans, rats, and mice	[52, 163, 164]
	Increased expression	Protection from ischemia-reperfusion (I/R) injury	Mouse heart	[165]
		Decreased glycolysis, increased oxygen consumption rate, and ATP levels	Old normal human fibroblasts	[162]
Drp1	Reduced expression	Development of cardiac dysfunction	Mouse heart	[166, 167]
		Attenuated diabetes-induced cardiac dysfunction	Streptozotocin- (STZ-) induced diabetic mice	[66]
		Protection against posttraumatic/diabetes-induced cardiac dysfunction	Adult rats	[168]
	Inhibition	Protection from cardiac hypertrophy and function after I/R injury or myocardial infarction	Mouse heart	[169, 170]
		Improved LV functions, reduced MI size	Mouse heart	[64]
		Protection from Dox-induced cardiac damage	H9c2	[65]
	Short-term induction in midlife	Prolonged lifespan	Drosophila melanogaster	[62]
PINK1	Increased expression	Increased cell senescence	Neonatal rat cardiomyocytes	[125]
	Activation	Improved mitochondrial function, decreased ROS production, decreased apoptosis	Mouse heart	[124]
Parkin	Increased expression	Prolonged lifespan	Drosophila melanogaster	[171]
		Decayed aging	Mouse	[172]
	Reduced expression	Impaired recovery of cardiac contractility	Mouse heart	[173]
FUNDC1	Abrogation	Sustained mitochondrial fission, cell death, and heart failure	Adult mice cardiac progenitor cells (CPCs)	[104]
	Increased expression	Increased mitophagy and reduced platelet activity, protection from I/R injury	Mouse	[125]
		Infarction area expansion and cardiac dysfunction following acute cardiac IR injury	Mouse	[85]
BNIP3	Suppressed activity	Stressed cardiomyocytes	Human heart	[174]
BECN1/Beclin1	Increased expression	Attenuated heart failure	Mouse heart	[60]
	Deceased interaction with BCL-2	Improved healthspan, prolonged longevity	Mutant mice	[175]
PCG-1*α*	Overexpression	Suppressed aging-induced mitophagy, improved mitochondria	Mouse skeletal muscle	[129]
